# Contamination Profile of Different Formulations of Silicone Oil Tamponade Before and After Intraocular Permanence for Rhegmatogenous Retinal Detachment

**DOI:** 10.1167/tvst.13.3.4

**Published:** 2024-03-11

**Authors:** Carlo Bellucci, Nicolò Riboni, Guido Ricciotti, Federico Spadini, Andrea Pasquali, Maurizio Rossi, Stefano Gandolfi, Erika Ribezzi, Enrico Marraffa, Federica Bianchi, Maria Careri, Paolo Mora

**Affiliations:** 1 Ophthalmology Unit, University Hospital of Parma, Parma, Italy; 2 Department of Chemistry, Life Sciences and Environmental Sustainability, University of Parma, Parma, Italy; 3 Department of Clinical and Experimental Medicine, University Hospital of Parma, Parma, Italy

**Keywords:** pars plana vitrectomy (PPV), silicone oil (SO) tamponade, gas chromatography-mass spectrometry-based analysis, contaminants, siloxanes, perfluorodecalin

## Abstract

**Purpose:**

The purpose of this study was to search for contaminants in silicone oil tamponades removed from eyes treated for retinal detachment, and to correlate chemical results with some clinical/functional parameters of the considered eyes.

**Methods:**

We examined a sequential cohort of eyes grouped according to the tamponade received: (1) Siluron2000 (S2), (2) RS-OIL ECS5000 (S5), and (3) Densiron Xtra (DX). Samples were collected at the beginning of the scheduled removal and analyzed by untargeted headspace gas-chromatography mass spectrometry (HS-GC-MS). Visual acuity and optic coherence tomography assessments were obtained before and after the tamponade removal.

**Results:**

Forty-one samples were analyzed: 22 belonging to the DX group, 13 to the S2 group, and 6 to the S5 group. For each group, a mixture of uninjected commercial preparation was analyzed as the reference. Different siloxanes and fluorinated compounds including perfluorodecalin (PFCL) were the most prevalent chemicals, found in 55% to 100% of the intraocular samples of the 3 groups. Some siloxanes were present also in the control matrices, whereas PFCL was only in the extracted tamponades. In the DX group, the concentration of hexamethylcyclotrisiloxane showed an inverse correlation trend with the duration of its permanence inside the eye (*P* = 0.054). Different alkanes, propanol, and acetaldehyde were identified only in the control matrices.

**Conclusions:**

Several contaminants including siloxanes were identified in the intraocular samples and in the control matrices. A time-related ocular uptake of some of these is conceivable. PFCL was also highly present but only in intraocular samples.

**Translational Relevance:**

After intraocular permanence silicone oils (SOs) have various unlabeled contaminants with some relevant differences with the commercial formulation chemical profile.

## Introduction

Rhegmatogenous retinal detachment (RRD) is a major vision-threatening disease with variable incidence ranging between 6 and 18 per 100,000 people.^1^ It develops from retinal tears, which can be related to different causing or predisposing conditions, such as trauma, retinal anomalies, cataract surgery, mainly in highly myopic eyes, or posterior vitreous detachment. Surgical management is required when separation of the neurosensory retina from the retinal pigment epithelium progresses. At present, pars plana vitrectomy (PPV) with different instrument gauges is the most adopted treatment. The surgical procedure is completed by introducing an intraocular tamponade, whose type depends on the clinical characteristics of the RRD. In general, tamponades can be either a self-dissolving air or gas mixture, or viscous compounds based on silicone oil (SO) formulations. In this latter instance, a further surgery is required after a variable time for intraocular tamponade removal. Several formulations of viscous tamponade are commercially available.

Among the complications associated with the use of SO, there is the occurrence of a sudden and severe loss of visual acuity.^2^ It mostly concerns eyes with prior complex retinal detachment, with or without macular involvement.^3^^–^^5^ Visual loss occurs more frequently shortly after SO removal but it has been reported even when the tamponade is still in the eye 1 to 5 months after its injection.^4^^–^^8^ Some microstructural or vascular anomalies have been described in eyes with SO tamponade; however, the etiology of this severe side effect is far from being fully understood. Some studies have demonstrated that low-molecular-weight components (LMWCs) can be present as contaminants in commercial tamponade formulations. These could be responsible for toxic effects on the retina.^9^ Moreover, the migration of tamponade particles contained in the vitreous chamber toward the adjacent inner retinal layers has also been postulated by in vivo optical coherence tomography (OCT) assessments.^10^

This study had the aim to identify and quantify possible unexpected, or otherwise undisclosed, compounds in tamponade samples extracted from previously vitrectomized eyes, and in the respective commercial formulations. The results of the chemical analyses on the ex vivo samples were correlated with the functional outcome of the study eyes, the time of tamponade intraocular permanence, and the OCT analysis of the variation in macular ganglion cell inner plexiform layer (GCIPL) thickness before and after removal of the tamponade.

## Materials and Methods

The study included eyes that underwent surgical extraction of SO tamponade via PPV in the ophthalmology unit of Parma University Hospital between November 2021 and December 2022. Patients were selected as part of an ongoing study on the loss of visual acuity associated with the use of SOs as tamponade (#1396/2020). The study adhered to the ethical guidelines outlined in the Declaration of Helsinki, and all participants provided written informed consent. The following inclusion criteria were considered when selecting the eyes for oil sample collection: recovery of a chart-assessable distance best-corrected visual acuity (BCVA; measured by Early Treatment Diabetic Retinopathy Study chart at 4 meters) after the intraocular placement of the tamponade, with the threshold arbitrary set at 20/100 in order to detect possible unexpected visual loss after tamponade removal; good control of intraocular pressure with or without topical medications; and absence of acute retinal vascular alterations or pathological changes in the funduscopic appearance of the optic disc during the intraocular permanence of the tamponade. These criteria were evaluated during regular follow-up visits scheduled at 1 week, 1 month, 3 months, and in the week preceding tamponade removal. The need for further re-attachment surgeries after the first PPV, or laser treatments during the first postoperative follow-up, were considered exclusion criteria. This was due to the potential to affect the chemical profile of the original tamponade. In addition, the absence of tamponade emulsification in the anterior and posterior chamber of the study eyes was clinically assessed.^11^

The thickness of the GCIPL, assessed via OCT (Optic Disc Cube 200 × 200 protocol of the Cirrus HD-OCT 5000; Carl Zeiss Meditec, Dublin, CA, USA) was re-measured after tamponade removal (3 ± 1 weeks). This was done to compare in each study eye the latter value with the one measured with the tamponade in situ, 1 week after its intraocular placement. To accept the OCT values for the study, one of the authors checked the quality of each scan and the correct identification of the area of interest by the instrument software.

The permanence time of tamponade inside the eyes was determined according to the indications provided for each commercial SO type, combined with possible case-sensitive clinical evidence. The collected samples were grouped based on the chemical characteristic of the chosen formulations: (1) Siluron2000 (Fluoron GmbH, Ulm, Germany) comprising a mixture of 5% polydimethylsiloxane (PDMS) with a shear viscosity of 2,500,000 cSt, and 95% PDMS with a shear viscosity of 1000 cSt (S2 group); (2) RS-OIL ECS5000 (Alchimia, Ponte San Nicolò, Italy), consisting of 100 % PDMS with a kinematic viscosity of 5000 cSt (S5 group); and (3) Densiron Xtra (Fluoron GmbH, Ulm, Germany) consisting of a mixture of ultra-purified PDMS (69.5%), 10% very high molecular weight silicone oil, and ultra-purified perfluorohexyloctane (DX group). For each group, a sample from a mixture of at least three commercial, non-injected preparations was utilized as blank matrix.

Chemical analyses were performed using an innovative method for untargeted headspace gas-chromatography-mass spectrometry (HS-GC-MS). The details of this technique are described in a recent dedicated publication.^12^

### Surgical Procedure

The first PPV for RRD management was performed via a 3-port, 23-gauge system with chandelier endoillumination. Phacoemulsification was initially performed if not achieved before. A perfluorodecalin (PFCL) commercial solution (HPF10; Alchimia, Ponte San Nicolò, Italy) was used during all surgeries to stabilize the posterior pole of the retina. It was carefully removed before completing the PPV by an indirect exchange method (PFCL – air – SO).

The second PPV for oil removal was always performed using a 23-gauge instrumentation. For each study eye, when the first cc of tamponade was suctioned into the extraction syringe the sample was immediately transferred in a sterile tube and frozen at −22°C until analysis and the tamponade removal completed according to standard procedure. Only during the sample aspiration, the intraocular pressure was preserved via air infusion. Oil sampling was always the first procedure performed, postponing any possible anterior chamber lavage or laser treatment reinforcement later during removal surgery.

### Statistical Analysis

Statistical analysis was performed using SPSS (version 28; SPSS Inc., Chicago, IL, USA). All data were presented as mean ± standard deviation (SD). Normality distribution was evaluated by performing Kolmogorov–Smirnov test. The 1-way analysis of variance (ANOVA) and the Kruskal–Wallis tests were used to compare data that were normally and non-normally distributed, respectively. Dunn–Bonferroni tests were performed for post hoc comparisons. A *P* value of < 0.05 was considered statistically significant.

## Results

Forty-two samples of tamponade were collected for chemical analysis, which was applicable in 41 samples. These samples were extracted from 41 eyes of as many patients (11 women and 30 men, mean age = 61 years, age range = 47–78 years). All cases originally had a macula of RRD and the time between diagnosis and first PPV ranged between 1 and 5 days.

Based on the type of tamponade, the samples were divided as follows: 22 eyes in the DX group; 13 eyes in the S2 group, and 6 eyes in the S5 group. The demographic data, the BCVA, and the intraocular pressure (IOP) pre- and post-tamponade removal in the 3 groups are summarized in Table 1. For these two latter parameters, the variations did not statistically differ neither between the different tamponade groups nor within every single group (i.e. between pre- and post-tamponade removal). No cases of remarkable visual loss (i.e. ≥ 2 Early Treatment Diabetic Retinopathy Study [ETDRS] lines in comparison to the BCVA assessed 1 week after tamponade placement) were observed at the end of the post-SO removal follow-up.

**Table 1.** Demographics and Clinical Data of Patients in the Three Study Groups

	DX (*n* = 25)	S2 (*n* = 13)	S5 (*n* = 6)	*P* Value
Age, y	62 ± 8	62 ± 10	58 ± 11	0.79
Sex (M/F)	13/9	11/2	5/1	0.08
BCVA PRE (LogMAR)	0.5 ± 0.3	0.4 ± 0.3	0.4 ± 0.3	0.71
BCVA POST in (LogMAR)	0.4 ± 0.3	0.5 ± 0.3	0.6 ± 0.3	0.37
IOP PRE (mm Hg)	14 ±5	15 ± 7	12 ± 5	0.12
IOP POST (mm Hg)	15 ± 5	16 ± 3	17 ± 6	0.22

BCVA, best-corrected visual acuity; DX, Densiron Xtra group; IOP, intraocular pressure; IQR, interquartile range; POST, after tamponade removal; PRE, before tamponade removal; S2, polydimethylsiloxane 2000 group; S5, polydimethylsiloxane 5000 group.

Data are presented as mean ± standard deviation.

The mean duration (±SD) of tamponade intraocular permanence in the eyes was 80 ± 36 days in the DX group, 99 ± 29 days in the S2 group, and 94 ± 39 days in the S5 group. The difference among the duration of intraocular permanence in the three tamponade groups was not statistically significant (*P* = 0.2).

Table 2 shows all the chemical compounds identified in at least one extracted sample; the table reports which of these compounds was also found in the respective control matrix obtained from commercial formulations. These unlabeled compounds belong to different chemical classes, namely alcohols, ketones, aldehydes, and saturated and unsaturated cyclic and linear hydrocarbons. Fluorinated and perfluorinated compounds were also identified in the extracted samples. In particular, the PFCL was identified in at least 50% of the samples regardless of the type of tamponade used.

**Table 2.** List of Chemicals Found in the Extracted Tamponades (Decreasing Prevalence) and in the Respective Blank Matrices for the Three Study Groups

CHEMICALS^a^	DX (Samples/22)	DX (Matrix)	S2 (Samples/13)	S2 (Matrix)	S5 (Samples/6)	S5 (Matrix)
Octamethylcyclotetrasiloxane	20	Y	12	Y	6	Y
Hexamethylcyclotrisiloxane	17	Y	7	Y	5	Y
Perfluorodecalin	14	–	8	–	3	–
Fluorinated compound (not assigned)	12	–	8	–	5	–
Decamethylcyclopentasiloxane	–	–	13	Y	6	Y
Cyclohexanone	4	–	11	–	4	–
Hydrocarbon (not assigned)	18	–	–	–	–	–
Dodecamethylcyclohexasiloxane	–	–	12	Y	6	Y
Tert-pentylcyclohexane	17	–	–	–	–	–
Hexane	16	Y	1	Y	–	Y
Acetone	12	Y	2	–	3	Y
Ketone (not assigned)	16	Y	–	–	–	–
Dodecanal	16	Y	–	–	–	–
2-Methylpentane	15	Y	–	Y	–	Y
Tetrahydrofuran	9	–	3	–	3	–
Tetradecafluoro-2-methylpentane	9	–	5	–	1	–
Pentafluoroethane	14	–	–	–	–	–
Siloxane (not assigned)	–	–	1	–	6	Y
Pentane	7	Y	–	Y	–	–
Sulfurane	–	–	2	–	4	–
3-Methylpentane	5	Y	–	Y	–	Y
Decamethyltetrasiloxane	–	–	–	–	4	Y
Perfluoromethylcyclohexane	2	–	1	–	–	–
Hexadecafluoroheptane	1	–	–	–	1	–
Tetradecafluorohexane	2	–	–	–	–	–
1-Undecyn-4-ol	2	–	–	–	–	–
Octamethyltrisiloxane	–	–	–	–	2	Y
2,4-Dimethyldecane	2	–	–	–	–	–
1,3-Pentadiene	1	–	–	–	–	–
2-Butene	1	Y	–	Y	–	Y
2-Methylundecanal	1	–	–	–	–	–
Ketone (not assigned)	1	–	–	–	–	–
Ester (not assigned)	1	–	–	–	–	–
Phenyl ether	–	–	–	–	1	–
Hexamethyldisiloxane	–	–	1	–	–	–
1-Propanol	–	Y	1	Y	–	Y
2-Butanone	–	Y	–	–	–	–
2-Methylbutane	–	Y	–	–	–	–
2-Pentanone	–	Y	–	–	–	–
Acetaldehyde	–	Y	–	–	–	–
Cyclohexane	–	Y	–	Y	–	Y
Methylcyclopentane	–	Y	–	Y	–	Y

DX, Densiron Xtra group; N, not present in the sample; S2, Polydimethylsiloxane 2000 group; S5, Polydimethylsiloxane 5000 group; Y, present in the sample.

a Compounds identified by comparison of their mass spectra with those stored in the NIST library and by comparison of calculated and tabulated retention indices.

Concerning LMWCs, we specifically addressed short-chain siloxanes such as hexamethyldisiloxane (L2), hexamethylcyclotrisiloxane (D3), octamethyltrisiloxane (L3), octamethylcyclotetrasiloxane (D4), decamethyltetrasiloxane (L4), decamethylcyclopentasiloxane (D5), and dodecamethylcyclohexasiloxane (D6).

The Figure shows for each tamponade group the percentage of samples in which such LMWCs were identified and whether they were also present in the commercial formulation. As a general comment, it can be stated that cyclic siloxanes were present in all the extracted samples and some of the investigated analytes were already present in the oils before the use. More precisely, D3 and D4 were detected in all groups in percentages > 50%; D6 was found in S2 and S5 groups; L3 and L4 were found only in the S5 group.

**Figure.**
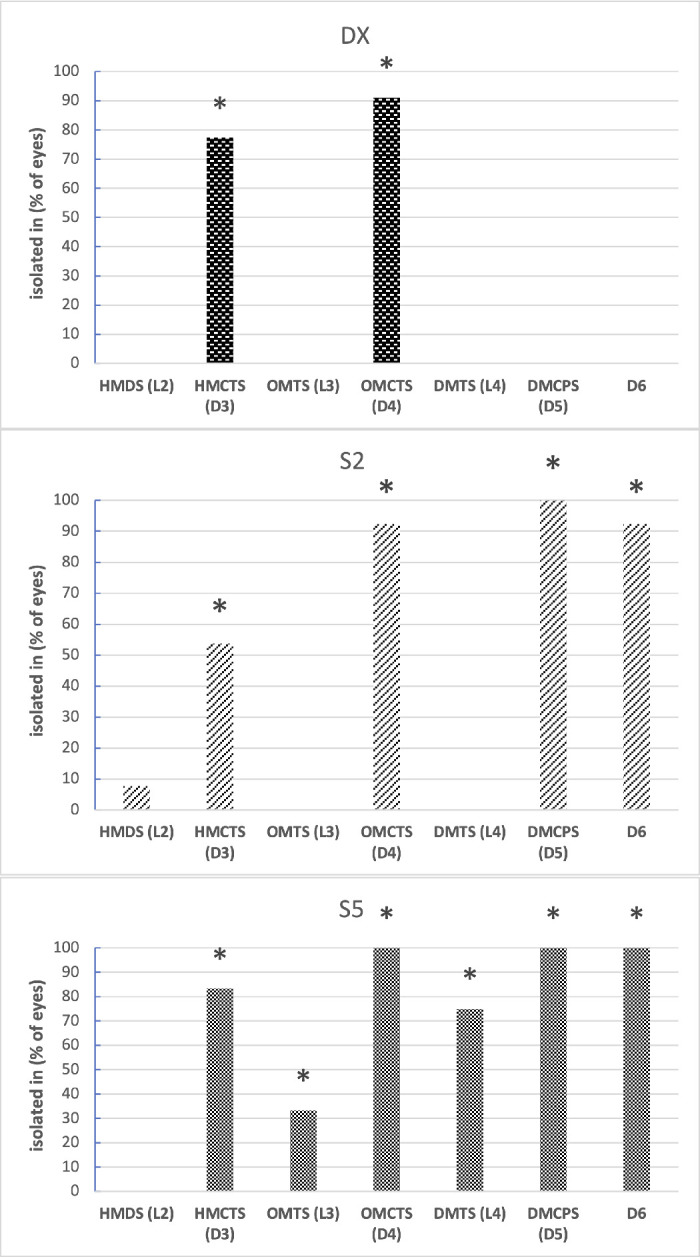
For each tamponade group, the plot shows the different types of siloxanes isolated (*horizontal axis*) and the percentage of eyes in which they were found (*vertical axis*). The *asterisk* indicates if the corresponding siloxane was present also in the blank matrix.

For the more widespread compounds, namely D3 and D4, it was possible to investigate the statistical correlation between their average concentration and the duration of ocular residence. As a trend, an inverse correlation was observed, reaching borderline statistical significance (*P* = 0.05) for D3 in the DX group.

The contamination rate (CR) of each tamponade group was also assessed; this parameter was obtained from the following calculation: CR = [number of samples with identified chemicals (any)/total number of eyes potentially involved] %. PFCL was excluded since it came from an intraoperative contamination given its absence in all the matrices. The CR was: 38.0% for the DX group, 39.6% for the S2 group, and 61.9% for the S5 group. Other substances including alkanes, propanol, and acetaldehyde (see the lower part of Table 2) were found in the control matrices of the three groups (chiefly in the DX) and in none of the extracted samples.

All included eyes underwent macular GCIPL measurements. It was possible to accept and compare OCT images from 31 eyes: 18 eyes in the DX group, 9 eyes in the S2 group, and 4 eyes in the S5 group (2 eyes with suitable quality were excluded for macular edema at the post-removal assessment). The small sample size allowed for a cumulative statistical comparison only. The mean GCIPL thickness was 59 ± 26 µm with intraocular tamponade, and 79 ± 16 µm after tamponade removal (*P* = 0.042).

## Discussion

The aim of this study was to investigate the presence of contaminants in different types of intraocular tamponade extracted from eyes treated via PPV for RRD. From eligible eyes, the authors analyzed the samples collected in the setting of the scheduled removal surgeries. According to our clinical practice, the largest group of samples comprised a high-density tamponade (DX), followed by medium-viscosity tamponade (S2) and high-viscosity tamponade (S5). The use of a recently validated untargeted HS-GC-MS-method allowed the detection of a variety of possible impurities, in particular low-molecular-weight impurities.^12^

In our cohort, a first noticeable finding was the presence of PFCL aliquots in a consistent proportion of samples of the three groups. We explain this by a probable incomplete aspiration of PFCL during the first PPV, despite the intra-operative removal maneuvers, always achieved the complete aspiration of any visible remnant. This finding confirms the importance of the research for an ideal intra-operative retinal stabilizer, for instance, with greater surface tension easing its whole and safe removal.

A second observation concerns the likely unprovoked contamination profile of the ex vivo samples. This would result from combining the spectrum of unlabeled detected compounds to the number of samples in which these chemicals were actually present. Referring to this parameter, that we named CR, the DX and the S2 groups were less affected (38.0% and 39.6%, respectively) than the S5 group (61.9%). This evidence, although unspecific, can be considered when dealing with the choice of the most suitable tamponade.

Concerning siloxanes, we remark the following observations. Cyclic compounds such as D3 and D4 were present at high rates in samples of each tamponade group (see the Fig.). For these compounds, it was therefore possible to outline a trend of an inverse statistic correlation between concentration in the ex vivo samples and duration of permanence inside the eye. These data may support the hypothesis of a progressive dispersion of the mentioned compounds in the closest ocular tissues. D5 and D6 were both present in almost all the samples belonging to S2 and S5 groups and in the relative blank matrix, whereas they were absent in the DX group/matrix. Likely, the DX formulation is more prone to be free from such contaminants. Linear siloxanes (L3 and L4) were found in lower rates of the sole S5 samples and relative matrix. Because all the siloxanes found in the samples were often detected in the respective matrix, it is reasonable to believe that contamination was already present in the commercial formulations.

Referring to the tamponades used for ocular surgery, the presence of LMWCs, including siloxanes, and their possible biological interaction with the surrounding tissues have been a debated matter. Products of cell metabolism, such us cholesterol and fatty acids, have been detected in silicone oil after intraocular permanence.^13^^,^^14^ A previous series verified the chemical stability of a high viscosity SO tamponed after prolonged intraocular permanence.^15^

The potential role of LMWCs in the development of toxic effects on ocular tissues during the intraocular presence of SO tamponade was first hypothesized in the 1980s, particularly in relation to the volatility of certain compounds present in tamponades, such as cyclosiloxanes. These cyclosiloxanes demonstrated the ability to diffuse into surrounding tissues.^9^ It is interesting to note that a recent measurement of intraocular temperature conducted in vivo found a temperature gradient, with the anterior part of the eye having a lower temperature and increasing closer to the retina.^16^ Moreover, eyes filled with SO tamponade had a higher mean temperature than eyes with vitreous humour or balanced salt solution. Another possible mechanism of interaction between the tamponade and retina could be related to the direct passage of emulsified SO droplets into the retinal layers and optic nerve head.^17^ This latter study hypothesized that emulsification and migration of SO typically occur within 3 months after the placement of the tamponade. LMWCs toxicity has then been proposed to be related to the migration of these small molecules into cells, due to their low viscosity and higher possibility of emulsification.^18^^–^^21^ The molecular geometry and weight of some compounds may contribute to the difference in cytotoxicity among the various LMWCs tested.^22^ A recent study excluded any in vitro direct toxic effect of LMWCs on human retinal pigment epithelial cells.^23^ The results, however, were not unanimously agreed upon.^24^

A further remarkable issue in our series was that different chemicals, including alkanes, propanol, and acetaldehyde, were identified in the control samples of each group but in none of those taken from the eyes (see the lower part of Table 2). This evidence may be incidental. However, there is a hypothesis that these substances, very volatile at room temperature and pressure, were not found in the in vivo samples as they are absorbed by the ocular tissues.

The OCT analysis did not show noticeable anomalies in our comprehensive cohort. However, the macular GCIPL thickness showed a significant increase after the tamponade removal (considering the 3 groups together) in comparison to the corresponding pre-removal values, as already reported in the literature.^25^^–^^27^ It is possible that the mechanical compression may not be well-tolerated by the retina of eyes with pre-existing metabolic impairments of the GCIPL. Therefore, the mere lack of an increase in macular GCIPL thickness between measurements with a tamponade agent and those after its removal may serve as a biomarker of retinal damage. The insufficiently large sample size would have made the use of multivariate models unwise, although potentially very interesting. This due to the substantial risk of compromising the statistical power and overfitting the model to the available data.

In conclusion, this study presents an original chemical characterization of three different types of SO tamponade agents after their residence within vitrectomized eyes. Regarding the presence of unlabeled compounds, that can be considered as contaminants, we remarked the following items.

1. A considerable number of samples in every tamponade group showed the presence of perfluorodecalin. We assign this finding to a near complete removal of PFCL during the first PPV.
2. A variety of compounds were identified in the extracted samples of the three tamponade groups and in the uninjected commercial formulations used as controls. The most present chemicals were cyclosiloxanes. For D3, a borderline significant inverse correlation was found between its concentration in the ex vivo samples and the length of time the tamponade remained into the eyes.
3. Different unlabeled chemicals were identified in the control samples of each group but in none of the in vivo samples.

These two latter evidences are compatible with a progressive dispersion of some substances present in the SO tamponade into ocular tissues. The relative narrowness of the sample cohort, also faced with the wide variety of compounds commercially available, require further investigation into the pathogenesis of the risks associated with the use of SO tamponade agents.
